# Loop analysis of blood pressure/volume homeostasis

**DOI:** 10.1371/journal.pcbi.1007346

**Published:** 2019-09-12

**Authors:** Bruno Burlando, Franco Blanchini, Giulia Giordano

**Affiliations:** 1 Department of Pharmacy (DIFAR), University of Genova, Genova, Italy; 2 Biophysics Institute, National Research Council (CNR), Genova, Italy; 3 Department of Mathematics, Computer Science and Physics, University of Udine, Udine, Italy; 4 Delft Center for Systems and Control, Delft University of Technology, Delft, The Netherlands; Stanford University, UNITED STATES

## Abstract

We performed a mathematical analysis of the dynamic control loops regulating the vasomotor tone of vascular smooth muscle, blood volume, and mean arterial pressure, which involve the arginine vasopressin (AVP) system, the atrial natriuretic peptide system (ANP), and the renin-angiotensin-aldosterone system (RAAS). Our loop analysis of the AVP-ANP-RAAS system revealed the concurrent presence of two different regulatory mechanisms, which perform the same qualitative function: one affects blood pressure by regulating vasoconstriction, the other by regulating blood volume. Both the systems are candidate oscillators consisting of the negative-feedback loop of a monotone system: they admit a single equilibrium that can either be stable or give rise to oscillatory instability. Also a subsystem, which includes ANP and AVP stimulation of vascular smooth muscle cells, turns out to be a candidate oscillator composed of a monotone system with multiple negative feedback loops, and we show that its oscillatory potential is higher when the delays along all feedback loops are comparable. Our results give insight into the physiological mechanisms ruling long-term homeostasis of blood hydraulic parameters, which operate based on dynamical loops of interactions.

## Author summary

The efficiency and resilience of our body are guaranteed by the presence of myriads of dynamic control loops that regulate fundamental vital functions. In this work, we studied the regulatory mechanisms that govern the interplay of vasoconstriction/vasodilation, blood volume and mean arterial pressure. We analysed the loops in the system and showed the presence of two coexisting mechanisms for blood pressure regulation, which perform the same qualitative function, conferring robustness to the system: one mechanism tunes vasoconstriction, the other blood volume. We showed that both systems are candidate oscillators: either they are stable or they oscillate regularly around their unique equilibrium. We analysed a subsystem that describes the stimulation of vascular smooth muscle cells due to the hormones arginine vasopressin (AVP) and atrial natriuretic peptide (ANP): also this system is a candidate oscillator ruled by multiple negative-feedback loops, and its potential for oscillations is higher when all the loops have similar delay. Our results cast light on the fundamental physiological phenomena that preserve the stable functioning of blood pressure and volume. This could have even wider relevance if other homeostasis and endocrine systems displayed similar features, with repercussions on the management of adverse homeostasis shifts like hypertension.

## Introduction

Various physiological aspects of mammal physiology are tuned on daily fluctuations depending on the activity of a molecular circadian clock located in the suprachiasmatic nucleus of the hypothalamus [1–3]. In addition, it has been shown that different organs, tissues, and isolated cells retain their own circadian clock in each level [4, 5], and moreover that the suprachiasmatic nucleus regulates body activities over multiple time scales [6–8]. Hence, circadian cycles seem specific adaptations of a more general tendency of metabolic and physiological processes to undergo periodic oscillations. This is shown, on different time and anatomical scales, e.g. by the serum levels of endocrine factors, and by myocardial or neuronal pacemakers. At the cellular level, well defined oscillatory patterns have been also described, e.g. cytosolic calcium dynamics occurring in electrically-coupled vascular smooth muscle cells that are thought to play a role in the spontaneous contractile mechanism of vasomotion [9]. Possible evidence of complex interacting loops can also be envisaged, e.g. in the heartbeat that exhibits continuous fluctuations with complex structure occurring on the top of a general oscillatory behaviour [10, 11].

Such a body of evidence suggests that the organism’s functioning could be described as a very complex aggregation and interaction of functional loops, while the term loopomics has been introduced as a comprehensive concept for loop-oriented analysis of physiological traits [12]. Loop systems would respond to mutual influences and to external stimuli able to modulate their activity, but the achievement of organism’s stability, i.e. homeostasis, should require that the loops are intrinsic oscillators, even though their mutual interaction could yield a very complex oscillatory behaviour with scale-invariant features [13, 14]. Such hypothesis could lead to a basic paradigm of the organism’s physiology and is worth being investigated by exploring the arrangement of loops and their possible unifying traits [15–20].

The endocrine system is particularly suitable for this kind of approach, given a number of data about the mutual influences of secretory cells that realise closed loops [21, 22]. Following this way, we carried out a mathematical analysis of the interplay between arginine vasopressin (AVP), atrial natriuretic peptide (ANP) and the renin-angiotensin-aldosterone systems (RAAS). Such a complex of agents operates on vascular smooth muscle cells and the renal regulatory systems of body salt and water content, thereby allowing a fine tuning of mean arterial pressure (MAP) under various conditions. Besides the relationships among these systems at the systemic level, a detailed analysis of signalling cascades elicited within smooth muscle cells was also done.

Given the difficulty of analysing complex neuroendocrine networks, the analysis is aimed at extracting loop systems and reducing them to essential traits by formal analysis, thereby obtaining mathematical objects that can be subsequently combined together in a wider analysis, thus allowing a stepwise approach to the modelling of wider domains of the organism. In particular, our loop analysis revealed that the system functioning relies on negative-feedback regulatory loops able to exhibit either stability (homeostasis) or persistent oscillations. In the overall system, two equivalent subsystems coexist that perform the same qualitative function, conferring robustness.

### AVP system

AVP is a peptide hormone whose main sites of production are magnocellular neurons of the hypothalamic supraoptic (SON) and paraventricular nuclei (PVN). AVP is transported along neuron axons to the posterior pituitary gland, where it is stored and ultimately released into the blood stream, inducing antidiuretic and vasoconstrictive effects [23]. SON and PVN nuclei receive inhibitory afferences from stretch receptors located in the left atrium, as well as from aortic arch, and carotid sinuses stretch receptors [24].

AVP acts on V2 receptors in renal collecting duct cells through a cAMP-dependent pathway, leading to increased water permeability, decreased urine excretion, and eventually causing rises in blood volume and pressure. Moreover, at higher concentrations, such as in hypovolaemia with decreasing arterial blood pressure, AVP also stimulates vascular smooth muscle cells, causing vasoconstriction and mean arterial pressure (MAP) rise [25].

Opposing to AVP effects on vascular smooth muscle and blood pressure, the release of atrial natriuretic peptide (ANP) results in a vasorelaxing effect and fall in cardiac output. Such a cross-talk between the two hormones is due to increased atrial distension that occurs in response to increased blood pressure (heart afterload), and increased central venous pressure and venous return (heart preload) [26].

While atrial stretch receptors stimulate ANP release, they also act to depress AVP release trough inhibitory stimuli. The circumventricular organs of the brain exert major control on AVP release following plasma osmolality rise. The subfornical organ (SFO), an element of the circumventricular system of the third cerebral ventricle, contains osmoreceptors that stimulate AVP release from SON and PVN nuclei [27]. Conversely, right atrial stretch receptors and aortic arc baroreceptors respond to blood pressure and volume rises via glossopharyngeal and vagus projections to the nucleus tractus solitarii (NTS) of the dorsal medulla oblongata. By this way, these afferent fibers affect the activity of SON and PVN neurons, eventually inhibiting AVP release [28, 29].

### ANP system

The ANP is secreted in the heart by atrial myocytes upon atrial stretching and systemic blood pressure rise [30]. ANP causes, among other effects, vasodilation by relaxing vascular smooth muscle [31]. The effect is most pronounced in the presence of elevated plasma concentrations of vasoconstrictor hormones, such as in advanced cardiac failure, since plasma angiotensin, AVP and other vasoconstrictors are elevated in that setting [32].

### RAAS system

Renin is produced by juxtaglomerular cells of the kidneys, which reside in the afferent arterioles of glomeruli. The release of renin is regulated by three primary mechanisms, a renal vascular baroreceptor, which responds to changes in renal perfusion pressure within the afferent arteriole, a tubular, macula densa-dependent sensor that measures distal tubular salt concentration in the filtrate, and renal sympathetic nerves. Low blood pressure in the afferent arteriole and low sodium chloride concentration in the tubule at the macula densa both stimulate renin release [33, 34]. According to the classic view of the renin-angiotensin cascade, renin acts as a peptidase converting the *α*-2-globulin angiotensinogen to angiotensin I, followed by conversion of this latter to angiotensin II (ANGII) by the angiotensin converting enzyme (ACE) [35].

Main ANGII effects occur through its binding to the G-protein-coupled AT1 receptor that triggers G_*q*/11_-dependent phospholipase C activation, followed by IP3-mediated intracellular Ca^2+^ rise. This mechanism mediates different ANGII effects, including among others, vascular smooth muscle contraction leading to blood pressure rise, and increased production of aldosterone from the adrenal zona glomerulosa [36]. It has also been shown that ANGII stimulates AVP release through the activation of AT1 receptors present in the SFO [37], while it inhibits renin release in juxtaglomerular cells through an increase of intracellular Ca^2+^ that overcomes cAMP stimulation of renin release [38].

Aldosterone is released from adrenal glands following ANGII production under conditions of hypotension, or elevated plasma K^+^ levels. Aldosterone mainly acts on distal nephron components, viz. distal convoluted tubule, connecting tubule, and collecting duct, by binding to the intracellular mineralcorticoid receptor (MR), a ligand-activated transcription factor whose main functional targets include the epithelial Na^+^ channel (ENaC), the renal outer medullary K^+^ channel (ROMK), and the serum- and glucocorticoid-regulated kinase (SGK). Major systemic effects of the aldosterone action on kidneys are changes in vascular tone due to increased Na^+^ reabsorption and enhanced excretion of excess K^+^ [39].

ANP release by atrial myocytes is linked to the RAAS system, but such connection is still incompletely clarified. However, it has been shown that ANP reduces renin and aldosterone secretion, sympathetic nerve activity, and renal tubular Na^+^ reabsorption [40, 41].

### Vascular smooth muscle cells

AVP acts on target organs by stimulating G-protein-coupled receptors (GPCRs), including V1 receptors (V1R) in vascular smooth muscle cells [42]. V1R activation triggers a signalling pathway, sequentially involving the G_*q*_ subunit alpha of trimeric G protein, the phospholipase C alpha (PLC*α*) and the ensuing inositol trisphosphate (IP3) production, leading to Ca^2+^ release from intracellular stores through IP3 receptors (IP3R) [43].

In smooth muscle cells, the rise in intracellular Ca^2+^ leads to the formation of a complex between Ca^2+^ and calmodulin (CaM) that activates myosin light chain kinase (MLCK). This latter phosphorylates myosin light chain (MLC), thus enhancing myosin activity and initiating actomyosin interaction [44]. On the other side, activated CaM also binds to the caldesmon peptide, thus removing its hindering effect on actomyosin interaction in relaxed smooth muscle, due to caldesmon binding to actin-tropomyosin [45]. Hence, different CaM actions coordinately result in promoting smooth muscle contraction.

ANP acts on target cells by activating NPRA and NPRB transmembrane receptors endowed with intracellular guanylyl cyclase activity, leading to increased cellular levels of cyclic guanosine monophosphate (cGMP) [46]. Thereafter, cGMP-dependent protein kinase (PKG) acts as a major mediator of ANP-induced smooth muscle relaxation, through the activation of myosin-light-chain phosphatase (MLCP) [47]. The extent of actomyosin interaction and ensuing smooth muscle contraction is determined by the balance between the activities of CaM-activated MLCK on one side, and MLCP on the other side [48]. MLCP dephosphorylates MLC, thereby contrasting MLCK activity on MLC, and relaxing the muscle [49].

Furthermore, there is also evidence that ANP acting through NPRA exerts an activating effect on the plasma membrane calcium ATPase pump (PMCA), leading to a reduction of intracellular Ca^2+^ levels, followed by CaM deactivation [50].

On the other hand, in smooth muscle cells AVP is known to activate RhoA, a member of the Rho family small GTPases, through the coupling of its GPCR receptor to G_12/13_ subunit and activation of guanine nucleotide exchange factor (GEF) [51]. RhoA becomes activated by GEF in a GTP-bound form, and in turn activates its downstream Rho-associated protein kinase (ROCK). This latter phosphorylates and inhibits MLCP, resulting in increased MLC phosphorylation and actomyosin contraction [52]. ROCK also phosphorylates MLC directly, causing more muscle contraction, and concomitantly activates the protein phosphatase 1 regulatory subunit 14A (CPI-17), a phosphorylation-dependent inhibitor of MLCP [53].

## Results

### Loop arrangement of the complete AAR system

**Fig 1:**
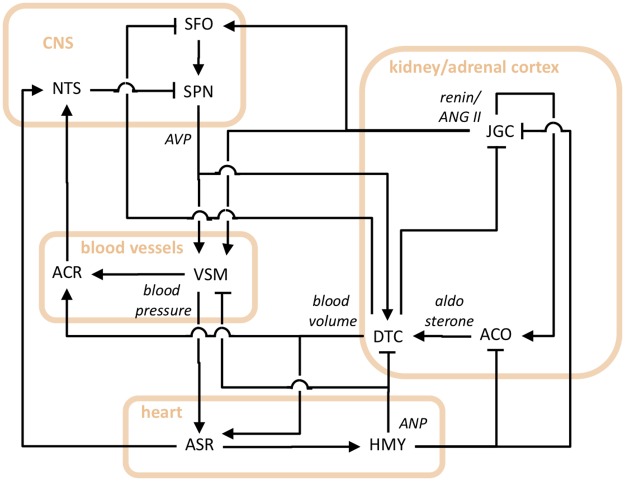
Diagram depicting antagonistic regulatory effects on vasoconstriction and blood pressure due to loop interrelationships among AVP, ANP, and RAAS neuroendocrine systems. Labels for functional agents are the following. ACO: adrenal cortex; ACR: aortic arc and carotid sinus stretch receptors; ASR: atrial stretch receptors; DTC: renal distal tubule and collecting duct; HMY: heart myocytes; JGC: juxtaglomerular cells; NTS: nucleus tractus solitarii; SFO: subfornical organ; SPN: supraoptic and paraventricular nuclei; VSM: vascular smooth muscle. Labels for effect mediators are the following. AVP: arginine vasopressin; ANP: atrial natriuretic peptide; ANGII: angiotensin II; CNS: central nervous system. Lines with arrow ending indicate activation; lines with butt ending indicate inhibition.

We built a model that describes dynamic control loops regulating the vasomotor tone of vascular smooth muscle, blood volume, and mean arterial pressure. The key players and their interactions are visually represented by the diagram of Fig 1.

The system is a completely-closed one, i.e. it has no free-terminal ends. It shows the interplay among AVP, ANP, and RAAS systems that occurs through their regulatory effects on vascular smooth muscle, blood volume and pressure. The activity and connections of baroreceptor (stretch receptors) and osmoreceptors are also included. The diagram consists of nodes, representing body systems producing physiological variations, and arcs connecting nodes, representing the mediators of these variations, viz. hormones, mechanical effects exerted by blood volume and pressure changes, and nerve signal conduction and neurotransmitter release. We denote this loop arrangement as the AAR (AVP-ANP-RAAS) system.

In the loop analysis of control systems, time constants and delays are essential parameters in the mathematical description of the system behaviour. Time constants are associated with the time intervals spanning between the stimulation and the activation of a functional agent at a given node. Delays correspond to the time intervals spanning between the activation of a functional agent at one node, and the stimulation of another functional agent at the downstream node. In the herein presented systems, the different processes can be grouped into four time-scale ranges: endocrine signals; mechanical effects operating on stretch receptors; nerve signal conduction; and intracellular signal transduction pathways.

The time response for endocrine signals is not always known with precision, but a wide complex of evidence on endocrine axes suggests that the time spanning from the secretion of a hormone to the response of its target cells should range between 30-60 min [54–56]. Data about the herein considered hormones are scarce, but pulsatile ANP secretion with a median frequency of 36 min has been found in healthy human subjects, thus being in line with the above estimates [57].

Also, an estimate for the time responses of stretch receptors to mechanical stimuli can be inferred from a study in the dog, where ANP secretion has been found to increase within 2.0 min of atrial distension, and to decline with cessation of atrial distension, with a half-time of 4.5 min [58]. These responses appear to be one order of magnitude faster than those of endocrine signals.

The time response along the nervous tracts of the system, depending on nerve signal conduction and synaptic interaction, can be estimated at below 1.0 min [59], while intracellular signal transduction pathways are even faster.

The presence of different processes characterised by time responses that differ of orders of magnitude enables mathematical simplifications relying on time-scale separation, which allowed us to rigorously analyse the complex interplay of interactions in the AAR systems.

#### Loop analysis of the AAR system

Consider the system represented in Fig 1. A structural loop analysis was performed to achieve the following main result: the overall control scheme can be functionally split into two redundant control systems, based on negative loops, which operate in parallel and qualitatively perform the same control action.

As discussed in detail in the Models and methods section, this result was achieved by

1. analysing the two control loops due to vascular smooth muscle and to renal distal tubule separately, which is justified since DTC and VSM do not mutually interact;
2. introducing proper simplifications based on time-scale separation arguments;
3. showing that both control loops can be seen as a negative feedback loop affecting a monotone system [60–65].

**Fig 2:**
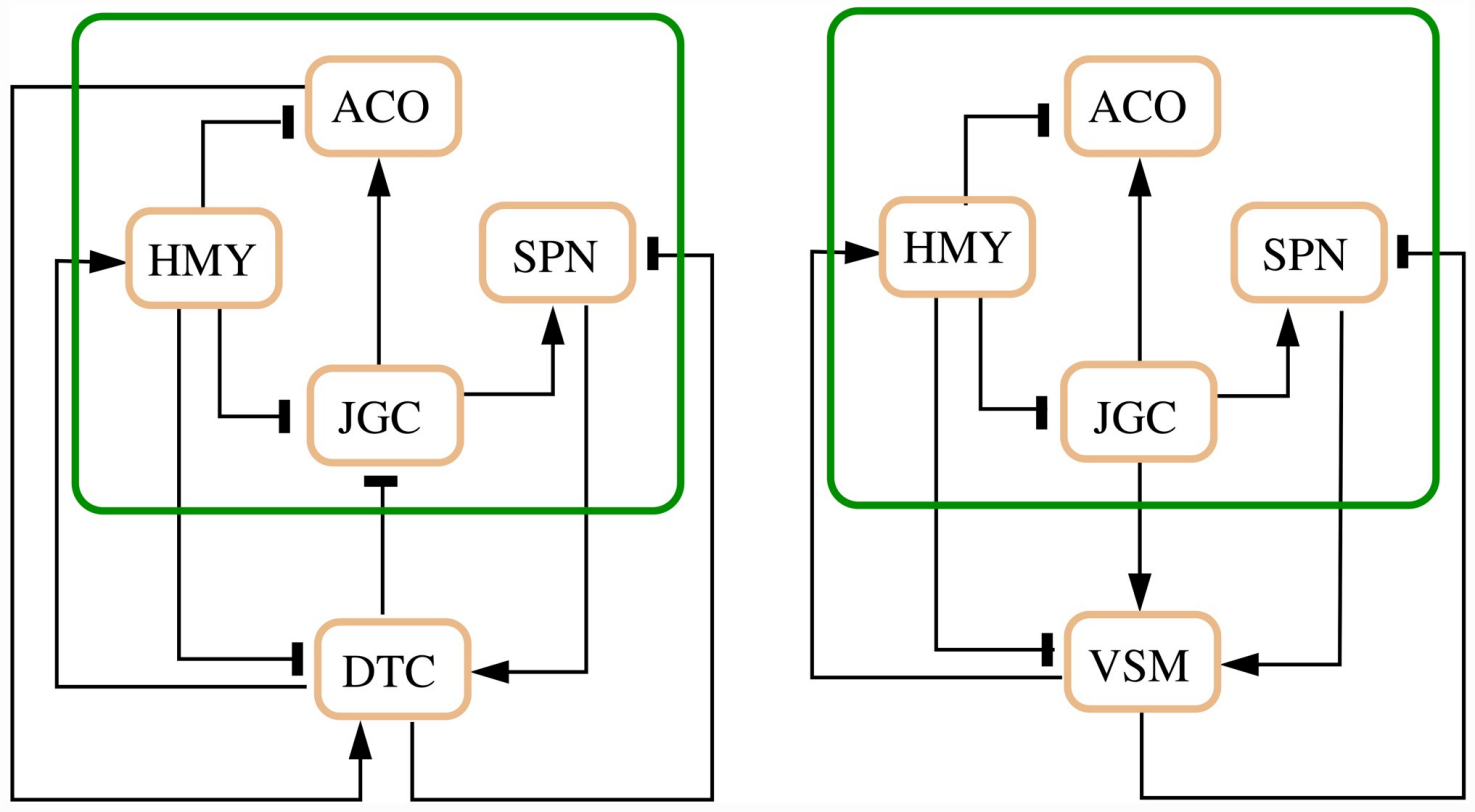
The two coexisting feedback schemes: The Renal Distal Tubule and Collecting Duct regulation (left) and the Vascular Smooth Muscle regulation (right).

The schemes describing the two coexisting regulation systems (which can be achieved from Fig 1 through the mathematical processing outlined in the three steps above—see also the Models and methods section below) are reported in Fig 2.

It is important to stress that each of the two systems in Fig 2, the one including DTC and the one including VSM, is a candidate oscillator according to the results in [15, 16], since it is the negative feedback of a monotone system (see the Models and methods section for details; [60–65]). Being a candidate oscillator, each of these systems admits a single equilibrium point (for each given choice of the parameter values), corresponding to all the variables being at steady state (homeostatic conditions); if the equilibrium becomes unstable, then persistent oscillations occur.

#### Influence analysis of the AAR system

An influence analysis showed that the two separate, but coexisting, regulation systems have the same qualitative behaviour and execute the same function, although the two schemes are structurally different. Indeed there is no one-to-one correspondence between the arcs. Precisely, the scheme in Fig 2, left, where the regulation is performed by DTC, has an additional activating arc, from ACO to DTC. Moreover, in the scheme in Fig 2, right, where the regulation is performed by VSM, the inhibitory arc from DTC to JGC is replaced by an activating arc from JGC to VSM. Remarkably, the influence matrices associated with the two systems are structurally consistent, as shown next.

The entry *M*_*ij*_ of the influence matrix *M* [66–68] (see also [20, 69–71]) expresses the sign of the steady-state variation of the *i*th variable of a dynamical system due to a persistent positive excitation caused by an external input applied to the dynamic equation of the *j*th variable. In our structural (parameter-free) analysis [66, 71], each entry of the influence matrix can assume the following values

$$\begin{array}{c} M_{ij}\in \left\{+,-,0,?\right\} \end{array}$$

where ‘+’ means that the sign of steady-state variation of the *i*th variable is always positive, regardless of the parameter values in the system; ‘−’ means that the sign of steady-state variation of the *i*th variable is always negative, regardless of the parameter values in the system; ‘0’ means that the sign of steady-state variation of the *i*th variable is always zero, regardless of the parameter values in the system; ‘?’ means that the sign of steady-state variation of the *i*th variable depends on the parameters.

The structural influence matrices corresponding to the systems in Fig 2 are

$$\begin{array}{c} M_{DTC}=\left[\begin{array}{cc} & \begin{array}{ccccc} \text{ACO} & \text{JGC} & \text{HMY} & \text{SPN} & \text{DTC} \end{array}\\ \begin{array}{c} \text{ACO}\\ JGC\\ \mathrm{HMY}\\ SPN\\ \text{DTC} \end{array} & \begin{array}{ccccc} + & ? & ? & - & -\\ - & + & ? & - & -\\ + & + & + & + & +\\ - & ? & ? & + & -\\ + & + & - & + & + \end{array} \end{array}\right]. \end{array}$$ (1)

and

$$\begin{array}{c} M_{VSM}=\left[\begin{array}{cc} & \begin{array}{ccccc} \text{ACO} & \text{JGC} & \text{HMY} & \text{SPN} & \text{VSM} \end{array}\\ \begin{array}{c} \text{ACO}\\ JGC\\ \mathrm{HMY}\\ SPN\\ \text{VSM} \end{array} & \begin{array}{ccccc} + & ? & - & - & -\\ 0 & + & - & - & -\\ 0 & + & + & + & +\\ 0 & ? & ? & + & -\\ 0 & + & - & + & + \end{array} \end{array}\right]. \end{array}$$ (2)

These matrices are qualitatively derived by considering only the sign matrices Σ_*DTC*_ and Σ_*VSM*_ associated with the schemes in Fig 2 (reported in the Models and methods section), since *M*_*DTC*_ = sign[adj(−Σ_*DTC*_)] and *M*_*VSM*_ = sign[adj(−Σ_*VSM*_)]; they can be efficiently computed by means of the algorithm proposed in [66].

Both the matrices are almost totally structurally determined: very few entries have a sign that depends on the parameters (and hence are ‘?’). Also, the two schemes are (weakly) consistent, because there is no contradiction between corresponding entries in the two structural influence matrices, apart from the first column, which is different because ACO does not affect any other key player in the VSM system, while in the DTC system it directly activates DTC, and thus indirectly affects all other key players.

### Loop arrangement of the AAV subsystem

**Fig 3:**
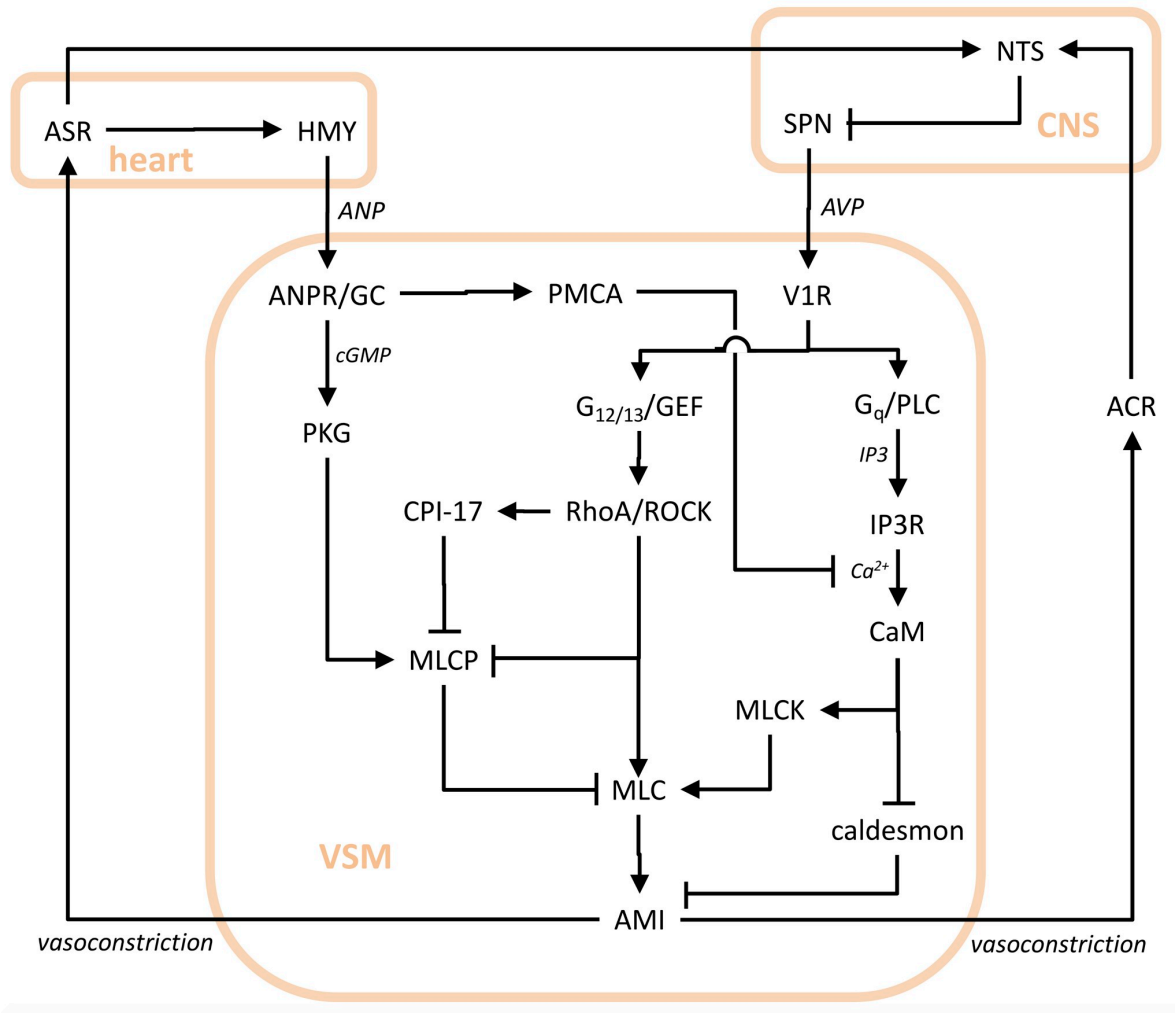
Diagram depicting antagonistic regulatory effects on vasoconstriction consisting of AVP and ANP endocrine systems acting on crosstalking signal transduction pathways of VSM, and stretch receptors closing the loops. Labels for functional agents are the following. AMI: actomyosin interaction; ANPR: atrial natriuretic peptide receptor; CaM: calmodulin; CPI-17: protein phosphatase 1 regulatory subunit 14A; GC: guanylate cyclase; GEF: guanine nucleotide exchange factor; G_*q*_: G_*q*_ alpha subunits of heterotrimeric G proteins; G_12/13_: G_12_/G_13_ alpha subunits of heterotrimeric G proteins; IP3R: inositol trisphosphate receptor; MLC myosin light chain; MLCK: myosin light chain kinase; MLCP: myosin-light-chain phosphatase; PKG: cGMP-dependent protein kinase; PLC: phospholipase C; PMCA: plasmamembrane Ca^2+^-ATPase; RhoA: Ras homolog gene family member A small GTPase; ROCK: Rho-associated protein kinase; V1R vasopressin receptor 1. Labels for effect mediators and anatomical districts are the following. cGMP: cyclic guanosine monophosphate; IP3: inositol trisphosphate. Other labels and line endings as in Fig 1.

After having examined the complete system, consisting of nodes acting at the systemic level, we made an attempt at combining the systemic and cellular levels. The nodes of the loops of our complete system (see Fig 1) represent cells that transfer signals from upstream to downstream elements by intracellular signal transduction pathways. Therefore, we analysed a system consisting of a subset of the above one, including ANP and AVP stimulation of vascular smooth muscle, a complex of crosstalks between AVP- and ANP-dependent signal transduction pathways operating within vascular smooth muscle cells, and stretch receptors closing loops onto AVP and ANP secretory systems. The system representation is shown in Fig 3. We denote this loop arrangement as the AAV (AVP-ANP-VSM) subsystem.

The choice for selecting vascular smooth muscle cells derives from the rather good knowledge of the interplay between AVP- and ANP-elicited pathways within these cells. Moreover, the choice of the AVP and ANP endocrine systems resides in their antagonistic effects, and the presumable similarity of their delays, since both involve only one slow endocrine step (time-limiting step), and a series of rapid intracellular processes, receptor responses, and nerve signal conduction steps.

#### Loop analysis of the AAV system

A structural loop analysis allowed us to obtain the following results, derived in the Models and Methods section:

- the AAV subsystem is monotone and evolves on a faster time scale than all other processes, hence it can be approximated as a single differential equation with first order dynamics;
- the subsystem is affected by three external negative feedback loops, which are reasonably modelled as due to delayed signals.

Hence, also this system is a candidate oscillator, as defined above [15, 16].

**Fig 4:**
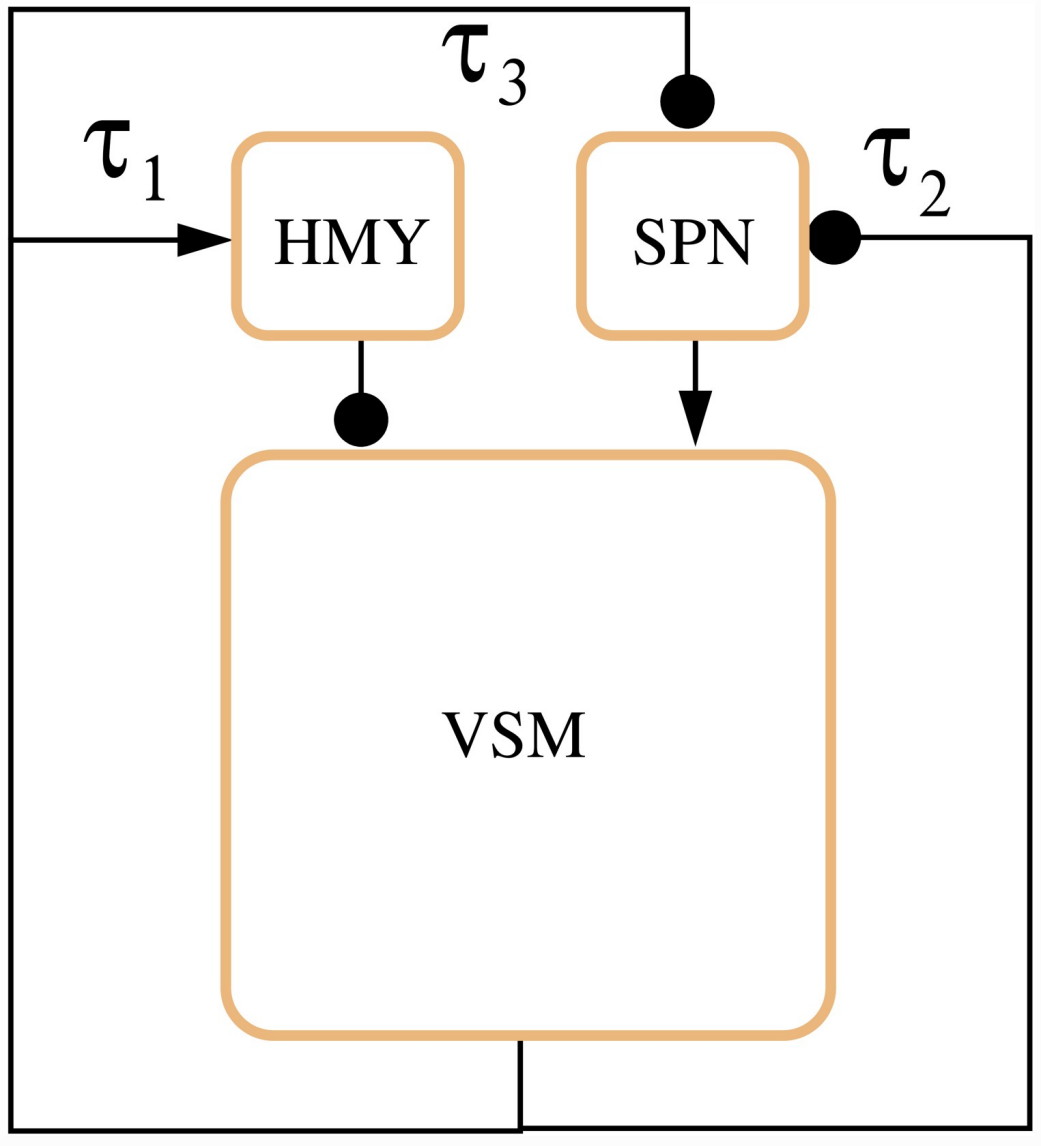
Visual representation of the reduced system in Eqs (3)–(5).

The system corresponds to the following dynamic model, associated with the graph in Fig 4.

$$\begin{array}{ccc} \Theta_{VSM}{\dot{x}}_{VSM}+x_{VSM} & = & g\left(x_{HMY}\right)+f\left(x_{SPN}\right) \end{array}$$ (3)

$$\begin{array}{ccc} \;x_{HMY} & = & f_{\tau_{1}}(x_{VSM}) \end{array}$$ (4)

$$\begin{array}{ccc} \;x_{SPN} & = & g_{\tau_{2}}(x_{VSM})+g_{\tau_{3}}(x_{VSM}) \end{array}$$ (5)

where Θ_*VSM*_ is the overall time constant of the VSM subsystem, *f* denotes increasing functions (associated with activation) and *g* decreasing functions (associated with inhibition), while the *τ*_*i*_’s denote delays. This simple dynamical system can effectively capture the essence of the vasoconstriction/vasodilation phenomenon.

In particular, to study its oscillatory properties, we analysed the linearised version of the system around an equilibrium point:

$$\begin{array}{ccc} \Theta_{VSM}\dot{y}\left(t\right)+y & = & -k_{1}u_{1}\left(t\right)+k_{2}\left[u_{2}\left(t\right)+u_{3}\left(t\right)\right] \end{array}$$ (6)

$$\begin{array}{ccc} u_{1}\left(t\right) & = & \mu_{1}y\left(t-\tau_{1}\right) \end{array}$$ (7)

$$\begin{array}{ccc} u_{2}\left(t\right) & = & -\mu_{2}y\left(t-\tau_{2}\right) \end{array}$$ (8)

$$\begin{array}{ccc} u_{3}\left(t\right) & = & -\mu_{3}y\left(t-\tau_{3}\right) \end{array}$$ (9)

where *y*, *u*_1_ and *u*_2_+ *u*_3_ are the variations of *x*_*VSM*_, *x*_*HMY*_ and *x*_*SPN*_ with respect to the equilibrium value, while *k*_*i*_ and *μ*_*i*_ denote the absolute value of the function derivatives.

To investigate the problem from a mathematical standpoint, we introduced a suitable oscillation-propensity index. As discussed in [19], for oscillations to occur:

- at least a negative loop must exist, associated with a delayed signal;
- the signal amplification through the negative loop, called loop gain, must be large enough.

Therefore, we took as oscillation-propensity index the minimum loop gain that is necessary for the onset of oscillations. The smaller this value, the more the system is prone to oscillations.

#### Delay analysis of the AAV subsystem

Through our delay analysis we showed that, when all the loops have approximately the same delay, so that they can be regarded as a single negative loop with delay, the system is prone to oscillations. Conversely, when the loops can have different delay, oscillations may be ruled out, because the resulting gain stability margin is larger when the delays are non-homogeneous.

First, we analysed the case of two different loop delays:

$$\begin{array}{c} \tau_{1}\;\text{and}\;\tau_{2}=\tau_{3}, \end{array}$$

which corresponds to assuming that the feedback signals *u*_2_ and *u*_3_, associated with the right atrium stretch receptors, have the same delay. This assumption is physiologically motivated by the fact that the interactions from HMY to VSM and from SNP to VSM (see Fig 4) are responsible for the largest part of the time delay.

Then, the system of Eqs (6)–(9) becomes

$$\begin{array}{c} \Theta_{VSM}\dot{y}\left(t\right)+y=-k_{1}\mu_{1}y\left(t-\tau_{1}\right)-k_{2}\left(\mu_{2}+\mu_{3}\right)y\left(t-\tau_{2}\right)=-py\left(t-\tau_{1}\right)-qy\left(t-\tau_{2}\right) \end{array}$$ (10)

This equation involves two negative-feedback delayed signals with gains *p* = *k*_1_*μ*_1_ and *q* = *k*_2_(*μ*_2_ + *μ*_3_), and delays *τ*_1_ and *τ*_2_, while the time constant is Θ_*VSM*_.

For small values of the gains *p* and *q*, the system is stable and does not oscillate. If we increase the gains above a certain threshold, oscillations will appear. The critical condition for the onset of oscillations is given by the equation

$$\begin{array}{c} j\omega \Theta_{VSM}+1+pe^{j\tau_{1}\omega }+qe^{j\tau_{2}\omega }=0 \end{array}$$ (11)

for some *p*, *q* and some *ω* > 0, where *j* is the imaginary unit and *ω* = 2*πf* is the pulsation corresponding to the oscillation frequency *f*.

**Fig 5:**
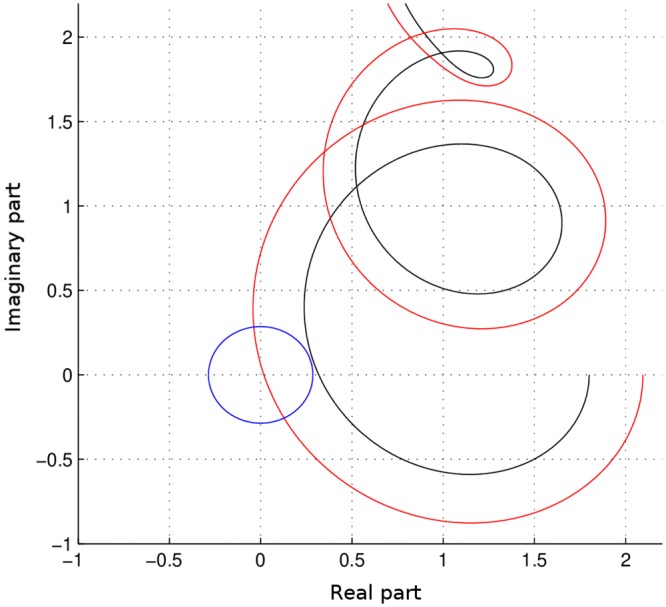
Function *jω*Θ_*VSM*_ + 1 + *pe*^*jτ*_1_*ω*^ + *qe*^*jτ*_2_*ω*^ plotted in the complex plane: Case with higher oscillation propensity (red), case with lower oscillation propensity (black). The radius of the blue circle centered in the origin and tangent to the curve is inversely proportional to the oscillation propensity.

In general, we can measure the oscillation propensity as follows. For given *p*, *q*, *τ*_1_, *τ*_2_ and Θ_*VSM*_, we consider the minimal distance of the curve *jω*Θ_*VSM*_ + 1 + *pe*^*jτ*_1_*ω*^ + *qe*^*jτ*_2_*ω*^ from the origin of the complex plane (see Fig 5). The oscillation propensity is defined as

$$\begin{array}{c} J^{*}=\frac{1}{\rho } \end{array}$$ (12)

where *ρ* is the radius of the circle tangent to the curve and centered at the origin (the blue circle in Fig 5, tangent to the black curve). Therefore, the smaller the radius (the closer the curve is to the origin), the larger the oscillation propensity. Note that, when (11) is satisfied for some $\bar{\omega }$, we have *ρ* = 0, hence J* = ∞.

Concerning the values of *p* and *q*, we obtained the following result.

**Proposition 1** *A necessary condition for* Eq (11) *to be satisfied is p* + *q* ≥ 1. *For p* + *q* = 1, *the equation has a solution (corresponding to ω* = *π*/*τ*) *if and only if* Θ_*VSM*_ = 0 *and all the delays are equal*, *τ*_1_ = *τ*_2_.

In fact, Eq (11) can only hold if *jω*Θ_*VSM*_ + 1 = −*pe*^*jτ*_1_*ω*^−*qe*^*jτ*_2_*ω*^, which is only true if the two moduli are equal. Since |*jω*Θ_*VSM*_ + 1| ≥ 1 if *ω* > 0, the modulus |*pe*^*jτ*_1_*ω*^ + *qe*^*jτ*_2_*ω*^|, which is at most *p* + *q*, must be larger than or equal to 1 as well.

The result indicates that the ideal situation for the onset of oscillations is that the two delays are close, *τ*_1_ ≈ *τ*_2_, and the time constant Θ_*VSM*_ is small. Hence, we can normalise the gains to get

$$\begin{array}{c} p+q=1 \end{array}$$ (13)

so that no oscillations are possible for Θ_*VSM*_ > 0.

**Fig 6:**
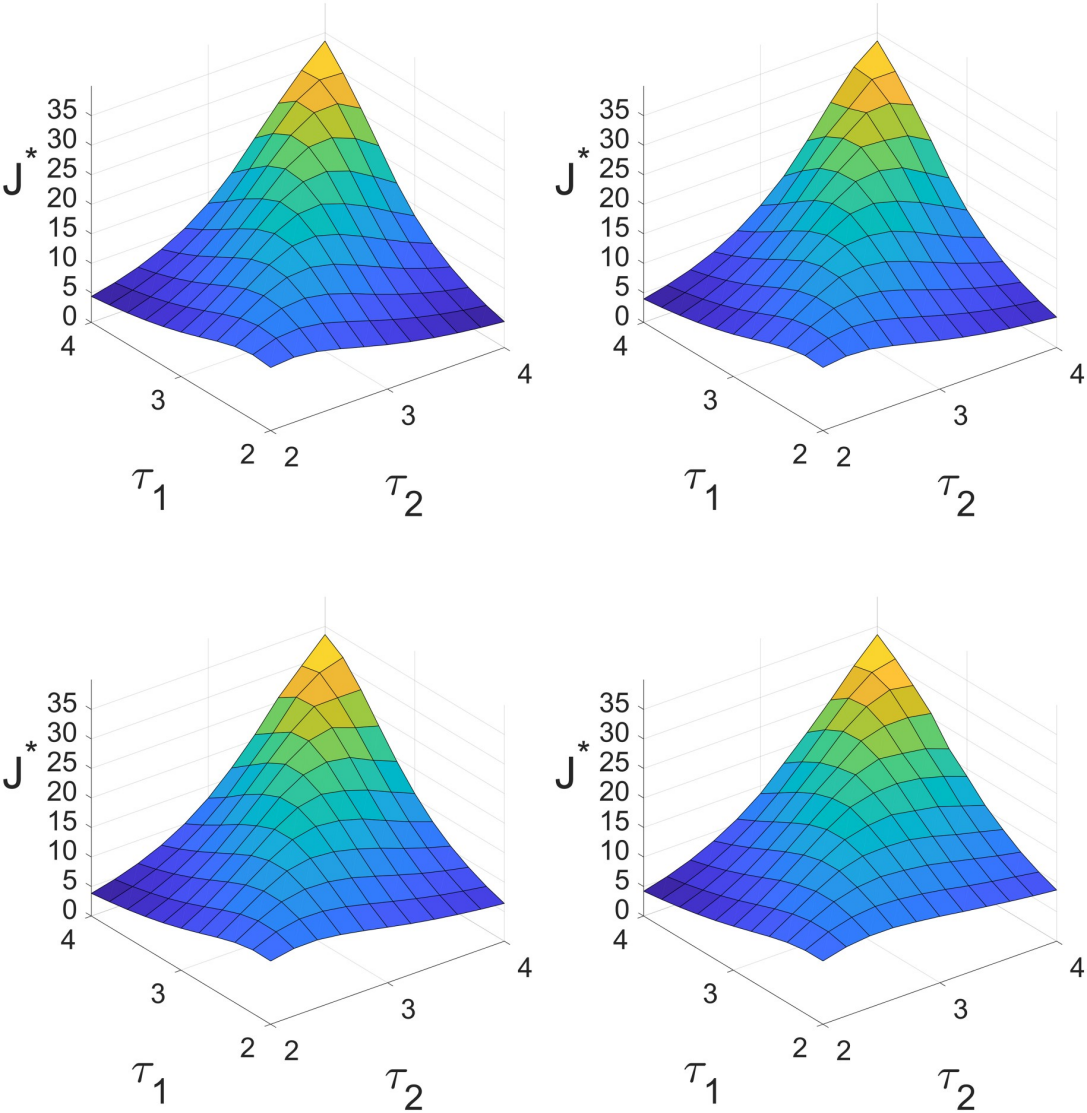
The oscillation propensity J* for Θ_*VSM*_ = 0.5 minutes as a function of the delays *τ*_1_ and *τ*_2_ in the range [2, 4] minutes, corresponding to *p* = 0.5 and *q* = 0.5 (top left), *p* = 0.4 and *q* = 0.6 (top right), *p* = 0.3 and *q* = 0.7 (bottom left), *p* = 0.2 and *q* = 0.8 (bottom right).

Then, given *p* and *q* satisfying Eq (13), and given Θ_*VSM*_, we can study the oscillation propensity as a function of the delay values *τ*_1_ and *τ*_2_. Fig 6 reports the results for Θ_*VSM*_ = 0.5 minutes and for various choices of the pair *p* and *q* = 1 − *p*, when *τ*_*i*_ are varied in the range [2, 4] minutes. The results show that the oscillation propensity is maximal when *τ*_1_ = *τ*_2_, namely when all the loop delays are equal, while it decays rapidly when the two delays are different.

An important consequence is the following: assuming that all the loops have approximately the same delay in normal conditions, then altering one of them by artificially changing its delay will hinder the oscillatory behaviour of the system.

## Discussion

Control loops are ubiquitous in biology at all scales, from individual cells to entire organisms, and are fundamental to rule the dynamic behaviour of living processes and to ensure homeostasis [72, 73]. Hence, living beings can be seen as fully integrated complexes of control systems, operating by loop dynamics. Biological and physiological mechanisms result from an extremely complex interplay of interactions; this complexity has been given theoretical interpretations in the framework of organisational closure [74, 75] and has been successfully analysed using system-theoretic and control-theoretic approaches [72, 73]. In complex biological networks, the presence of network motifs [76, 77] is fundamental to explain important behaviours; one of the most recurrent network motifs is the so-called feed-forward loop [73, 78]. Such a network analysis has been carried out not only at the cellular level, but also at the organismal level, leading to network physiology [79–81], which aims at explaining physiological functions based on the topology of the network of interactions.

In particular, the loop structures that can be found in the complex networks of dynamical interactions ruling life seem crucial to enable life-preserving dynamic behaviours in biology and physiology, and the functioning of each organism appears in fact as the result of complex aggregations and interactions of functional loops.

The well-documented AVP-ANP-RAAS endocrine control of body fluids and blood pressure was therefore analysed using mathematical tools, to highlight the loop arrangement and its dynamic function. The loop analysis of the whole system (AAR) shows that it can be split into two coexisting dynamic systems, which contain alternatively the VSM and DTC functional agents (as loop nodes), thereby exerting their effects on blood pressure and blood volume, respectively. The influence matrix analysis shows that the two systems are qualitatively equivalent in that they perform the same control function, even though the physiological mechanisms are different. Moreover, the loop analysis shows that both the VSM and the DTC loop systems are candidate oscillators having a single equilibrium point, which can be either stable or yield persistent oscillations under instability conditions. Also the AAV subsystem, combining functional agents acting at cellular and systemic scales, can be described as a candidate oscillator, whose propensity to exhibiting actual sustained oscillations is higher when the delays of all the loops in the subsystem are comparable.

Hence, our mathematical analysis suggests that the physiological mechanisms regulating long-term homeostasis of blood hydraulic parameters are arranged into a complex of equivalent loop systems, consisting of candidate oscillators with a single equilibrium point. Also, the whole system can be split into two systems displaying essentially the same functioning, an apparent redundancy that could offer alternatives for coping with accidental defaults, similar to the well-known, alternative kidney-lung regulation of blood pH [82]. Of course, our model must be seen as functionally coupled to other body systems, like the sympathetic and parasympathetic neurovegetative branches [83], while the interaction of multiple negative loop systems could be at the basis of complex oscillatory behaviours, with stochastic flavour, detected in the time course of physiological processes [84].

It is worth stressing that all the results we derived are completely independent of the exact functional expressions associated with activating and inhibitory interactions in the loop dynamics, and of the exact function parameters. Hence we can be sure that they hold for any system with this qualitative structure, even when we lack precise quantitative information.

Future research will be oriented to understanding if other homeostasis and endocrine systems display the same features, in order to possibly formulate a general paradigm in terms of loop dynamics. This achievement could have repercussions on the study and management of adverse homeostasis shift, e.g. due to chronic diseases like hypertension, frequently causing premature death [85].

## Models and methods

### Mathematical model of the AAR system

To build the dynamical system associated with the scheme in Fig 1, we model the activating and inhibitory interactions in terms of monotonic functions: we denote by

- *f* an activation function, monotonically increasing in its argument(s),
- *g* an inhibition function, monotonically decreasing in its argument(s),
- *h* an activation/inhibition function, increasing in the first argument and decreasing in the second.

Common examples are, for instance, the Hill-type functions:

$$\begin{array}{c} f\left(\left[X\right]\right)=\alpha \frac{{\left[X\right]}^{p}}{1+\beta {\left[X\right]}^{p}},\;\;g\left(\left[X\right]\right)=\gamma \frac{1}{1+\delta {\left[X\right]}^{p}},\;\;h\left(\left[X\right],\left[Y\right]\right)=\sigma \frac{{\left[X\right]}^{p}}{1+\epsilon {\left[X\right]}^{p}+\eta {\left[Y\right]}^{p}}, \end{array}$$ (14)

where [*X*] and [*Y*] represent the concentration of chemical species *X* and *Y*, *p* is the Hill coefficient (typically a cooperativity index), while *α*, *β*, *γ*, *δ*, *σ*, *ϵ* and *η* are positive coefficients. Note that the functions in Eq (14) are just examples of possible functional expressions, but our results are totally independent of the exact functional expressions associated with activations and inhibitions, and of the function parameters. Hence, we do not use any information beyond the fact that *f* and *g* are monotonic functions, increasing and decreasing respectively. Then, the system in Fig 1 is qualitatively described by the following differential equations:

$$\begin{array}{ccc} \Theta_{ACO}\frac{d}{dt}\left[ACO\right]+\left[ACO\right] & = & f([JGC])+g([HMY]) \end{array}$$ (15)

$$\begin{array}{ccc} \Theta_{JGC}\frac{d}{dt}\left[JGC\right]+\left[JGC\right] & = & g([HMY])+g([DTC]) \end{array}$$ (16)

$$\begin{array}{ccc} \Theta_{HMY}\frac{d}{dt}\left[HMY\right]+\left[HMY\right] & = & f([ASR]) \end{array}$$ (17)

$$\begin{array}{ccc} \Theta_{SPN}\frac{d}{dt}\left[SPN\right]+\left[SPN\right] & = & g([NTS])+f([SFO]) \end{array}$$ (18)

$$\begin{array}{ccc} \Theta_{DTC}\frac{d}{dt}\left[DTC\right]+\left[DTC\right] & = & f([ACO])+g([HMY])+f([SPN]) \end{array}$$ (19)

$$\begin{array}{ccc} \Theta_{VSM}\frac{d}{dt}\left[VSM\right]+\left[VSM\right] & = & f([JGC])+g([HMY])+f([SPN]) \end{array}$$ (20)

$$\begin{array}{ccc} \Theta_{ASR}\frac{d}{dt}\left[ASR\right]+\left[ASR\right] & = & f([DTC])+f([VSM]) \end{array}$$ (21)

$$\begin{array}{ccc} \Theta_{ACR}\frac{d}{dt}\left[ACR\right]+\left[ACR\right] & = & f([DTC])+f([VSM]) \end{array}$$ (22)

$$\begin{array}{ccc} \Theta_{NTS}\frac{d}{dt}\left[NTS\right]+\left[NTS\right] & = & f([ASR])+f([ACR]) \end{array}$$ (23)

$$\Theta_{SFO}\frac{d}{dt}[SFO]+[SFO]=f([JGC])+g([DTC])$$ (24)

This system of differential equations can be simplified in view of time-scale separation arguments, since the time constants Θ_*ACR*_, Θ_*ASR*_, Θ_*NTS*_ and Θ_*SFO*_ have the order of magnitude of few seconds or minutes, while all the others are of several (15 or more) minutes. Therefore we neglect the differential Eqs (21)–(24) by assuming

$$\begin{array}{c} \Theta_{ASR}=\Theta_{ACR}=\Theta_{NTS}=\Theta_{SFO}=0 \end{array}$$ (25)

and in Eqs (15)–(20) we substitute the expressions:

$$\begin{array}{ccc} \; & & f\left(\left[ASR\right]\right)=f(f([DTC])+f([VSM]))=f\left(\left[DTC\right],\left[VSM\right]\right) \end{array}$$ (26)

$$\begin{array}{ccc} \; & & g\left(\left[NTS\right]\right)=g(f([ASR])+f([ACR]))=g\left(\left[ASR\right],\left[ACR\right]\right)=g\left(\left[DTC\right],\left[VSM\right]\right) \end{array}$$ (27)

$$\begin{array}{ccc} \; & & f\left(\left[SFO\right]\right)=f(f([JGC])+g([DTC]))=h^{+-}\left(\left[JGC\right],\left[DTC\right]\right) \end{array}$$ (28)

where we exploit the fact that the composition of increasing functions is increasing, the composition of an increasing and a decreasing function is decreasing, and the composition of two functions, one increasing and one decreasing, with an increasing function produces a function *h*^+ −^ that is increasing in the first argument and decreasing in the second. The new system of equations becomes

$$\begin{array}{ccc} \Theta_{ACO}\frac{d}{dt}\left[ACO\right]+\left[ACO\right] & = & f([JGC])+g([HMY]) \end{array}$$ (29)

$$\begin{array}{ccc} \Theta_{JGC}\frac{d}{dt}\left[JGC\right]+\left[JGC\right] & = & g([HMY])+g([DTC]) \end{array}$$ (30)

$$\begin{array}{ccc} \Theta_{HMY}\frac{d}{dt}\left[HMY\right]+\left[HMY\right] & = & f([DTC],[VSM]) \end{array}$$ (31)

$$\begin{array}{ccc} \Theta_{SPN}\frac{d}{dt}\left[SPN\right]+\left[SPN\right] & = & g\left(\left[DTC\right],\left[VSM\right]\right)+h^{+-}\left(\left[JGC\right],\left[DTC\right]\right) \end{array}$$ (32)

$$\begin{array}{ccc} \Theta_{DTC}\frac{d}{dt}\left[DTC\right]+\left[DTC\right] & = & f([ACO])+g([HMY])+f([SPN]) \end{array}$$ (33)

$$\begin{array}{ccc} \Theta_{VSM}\frac{d}{dt}\left[VSM\right]+\left[VSM\right] & = & f([JGC])+g([HMY])+f([SPN]) \end{array}$$ (34)

The interaction matrix associated with the system, which reports the signs of the entries of the system Jacobian matrix (hence we basically associate a “+” with *f*, a “−” with *g*, and a “+” and a “−” with *h*^+−^), is then

$$\begin{array}{c} \Sigma =\left[\begin{array}{cc} & \begin{array}{cccccc} \text{ACO} & \text{JGC} & \text{HMY} & \text{SPN} & \text{DTC} & \text{VSM} \end{array}\\ \begin{array}{c} \text{ACO}\\ JGC\\ \mathrm{HMY}\\ SPN\\ \text{DTC}\\ \text{VSM} \end{array} & \begin{array}{cccccc} - & + & - & 0 & 0 & 0\\ 0 & - & - & 0 & - & 0\\ 0 & 0 & - & 0 & + & +\\ 0 & + & 0 & - & - & -\\ + & 0 & - & + & - & 0\\ 0 & + & - & + & 0 & - \end{array} \end{array}\right] \end{array}$$ (35)

By changing sign to the third variable, [*HMY*], we obtain

$$\tilde{\Sigma }=\left[\begin{array}{ccc} & \begin{array}{cccc} \text{ACO} & \text{JGC} & -\text{HMY} & \text{SPN} \end{array} & \begin{array}{cc} \text{DTC} & \text{VSM} \end{array}\\ \begin{array}{c} \text{ACO}\\ \text{JGC}\\ -\text{HMY}\\ \text{SPN} \end{array} & \begin{array}{cccc} - & + & + & 0\\ 0 & - & + & 0\\ 0 & 0 & - & 0\\ 0 & + & 0 & - \end{array} & \begin{array}{cc} 0 & 0\\ - & 0\\ - & -\\ - & - \end{array}\\ \begin{array}{c} \text{DTC}\\ \text{VSM} \end{array} & \begin{array}{cccc} + & 0 & + & +\\ 0 & + & + & + \end{array} & \begin{array}{cc} - & 0\\ 0 & - \end{array} \end{array}\right]$$ (36)

where we can see that all the interactions among the first four variables are cooperative: they are all associated with activations.

Therefore, the original system is equivalent, up to a state transformation where the sign of some variables is changed, to a cooperative system. Hence, it is a monotone system [60–65], characterised by a neat order-preserving behaviour that guarantees interesting properties. The fact that the overall system is the negative feedback loop of a monotone system implies that it admits a single equilibrium point, achieved when all the variables are at steady state [15, 16].

**Proposition 2** *The variables* [*ACO*], [*JGC*], [*HMY*] *and* [*SPN*] *form an input-output monotone subsystem, which is affected by two negative feedback loops*, *one due to* [*DTC*] *and one due to* [*VSM*].

In fact, both [*DTC*] and [*VSM*] are activated by the variables in the subsystem, which in turn they inhibit. Note that [*DTC*] and [*VSM*] do not interact with each other.

In view of these considerations, we can analyse separately the effect of the renal distal tubule [*DTC*] and the vascular smooth muscles [*VSM*]. They both can be considered as exerting a control action on the monotone subsystem including the variables [*ACO*], [*JGC*], [*HMY*] and [*SPN*]: the two loop schemes are in Fig 2.

The interaction matrices associated with the two schemes are:

$$\begin{array}{c} M_{DTC}=\left[\begin{array}{cc} & \begin{array}{ccccc} ACO & JGC & HMY & SPN & DTC \end{array}\\ \begin{array}{c} ACO\\ JGC\\ HMY\\ SPN\\ DTC \end{array} & \begin{array}{ccccc} - & + & - & 0 & 0\\ 0 & - & - & 0 & -\\ 0 & 0 & - & 0 & +\\ 0 & + & 0 & - & -\\ + & 0 & - & + & - \end{array} \end{array}\right] \end{array}$$ (37)

and

$$\begin{array}{c} M_{VSM}=\left[\begin{array}{cc} & \begin{array}{ccccc} ACO & JGC & HMY & SPN & VSM \end{array}\\ \begin{array}{c} ACO\\ JGC\\ HMY\\ SPN\\ VSM \end{array} & \begin{array}{ccccc} - & + & - & 0 & 0\\ 0 & - & - & 0 & 0\\ 0 & 0 & - & 0 & +\\ 0 & + & 0 & - & -\\ 0 & + & - & + & - \end{array} \end{array}\right] \end{array}$$ (38)

where the entries + and − denote, respectively, an arbitrary positive and negative value; the exact values depend on the system parameters and are unknown.

Note that each of the two systems, *DTC* and *VSM*, is a candidate oscillator according to the results in [15, 16], since it is the negative feedback of a monotone system. Being a candidate oscillator, each of these systems admits a single equilibrium point, corresponding to all the variables being at steady state (homeostatic conditions); if the equilibrium becomes unstable, then persistent oscillations occur.

Based on Σ_*DTC*_ and Σ_*VSM*_ (note that det[−Σ_*DTC*_] and det[−Σ_*VSM*_] are structurally positive, regardless of the signed values of the entries), the structural influence matrices corresponding to the two schemes in Fig 2 can be efficiently computed based on the algorithm proposed in [66], and are reported in the Results section.

### Mathematical model of the AAV subsystem

Consider the ensemble of intra- and inter-cellular loops regulating vasoconstriction and vasodilation through vascular smooth muscle cell contraction, visually represented by the diagram in Fig 3. We label the variables as *x*_1_ = [AMI], *x*_2_ = [caldesmon], *x*_3_ = [MLC], *x*_4_ = [MLCP], *x*_5_ = [MLCK], *x*_6_ = [CaM], *x*_7_ = [PKG], *x*_8_ = [CPI-17], *x*_9_ = [RhoA/ROCK], *x*_10_ = [IP3R], *x*_11_ = [G_*q*_/PLC], *x*_12_ = [G_12/13_/GEF], *x*_13_ = [PMCA], *x*_14_ = [ANPR/GC], *x*_15_ = [V1R], *x*_16_ = [HMY], *x*_17_ = [SPN], *x*_18_ = [NTS], *x*_19_ = [ASR], *x*_20_ = [ACR]. Let us denote the time derivative of *x*_*i*_ as ${\dot{x}}_{i}=\frac{d}{dt}x_{i}$ and the associated time constant as Θ_*i*_.

We adopt the same conventions as in the previous model to denote activating and inhibitory interactions. Moreover, we denote by *f*_*τ*_([*X*](*t*)) and *g*_*τ*_([*X*](*t*)), respectively, the functions *f* and *g* of the variable [*X*] delayed of a time interval *τ*:

$$\begin{array}{c} f_{\tau }\left(\left[X\right]\left(t\right)\right)=f\left(\left[X\right]\left(t-\tau \right)\right),\;g_{\tau }\left(\left[X\right]\left(t\right)\right)=g\left(\left[X\right]\left(t-\tau \right)\right). \end{array}$$

Then, the system in Fig 3 is qualitatively described by the following differential equations:

$$\begin{array}{ccc} \Theta_{1}{\dot{x}}_{1}+x_{1} & = & g\left(x_{2}\right)+f\left(x_{3}\right) \end{array}$$ (39)

$$\begin{array}{ccc} \Theta_{2}{\dot{x}}_{2}+x_{2} & = & g(x_{6}) \end{array}$$ (40)

$$\begin{array}{ccc} \Theta_{3}{\dot{x}}_{3}+x_{3} & = & g\left(x_{4}\right)+f\left(x_{5}\right)+f\left(x_{9}\right) \end{array}$$ (41)

$$\begin{array}{ccc} \Theta_{4}{\dot{x}}_{4}+x_{4} & = & f\left(x_{7}\right)+g\left(x_{8}\right)+g\left(x_{9}\right) \end{array}$$ (42)

$$\begin{array}{ccc} \Theta_{5}{\dot{x}}_{5}+x_{5} & = & f(x_{6}) \end{array}$$ (43)

$$\begin{array}{ccc} \Theta_{6}{\dot{x}}_{6}+x_{6} & = & h(x_{10},x_{13}) \end{array}$$ (44)

$$\begin{array}{ccc} \Theta_{7}{\dot{x}}_{7}+x_{7} & = & f(x_{14}) \end{array}$$ (45)

$$\begin{array}{ccc} \Theta_{8}{\dot{x}}_{8}+x_{8} & = & f(x_{9}) \end{array}$$ (46)

$$\begin{array}{ccc} \Theta_{9}{\dot{x}}_{9}+x_{9} & = & f(x_{12}) \end{array}$$ (47)

$$\begin{array}{ccc} \Theta_{10}{\dot{x}}_{10}+x_{10} & = & f(x_{11}) \end{array}$$ (48)

$$\begin{array}{ccc} \Theta_{11}{\dot{x}}_{11}+x_{11} & = & f(x_{15}) \end{array}$$ (49)

$$\begin{array}{ccc} \Theta_{12}{\dot{x}}_{12}+x_{12} & = & f(x_{15}) \end{array}$$ (50)

$$\begin{array}{ccc} \Theta_{13}{\dot{x}}_{13}+x_{13} & = & f(x_{14}) \end{array}$$ (51)

$$\begin{array}{ccc} \Theta_{14}{\dot{x}}_{14}+x_{14} & = & f(x_{16}) \end{array}$$ (52)

$$\begin{array}{ccc} \Theta_{15}{\dot{x}}_{15}+x_{15} & = & f(x_{17}) \end{array}$$ (53)

where *x*_16_ and *x*_17_ are external inputs and *x*_1_ is the output, coupled in a feedback loop with the differential equations describing the effect of the heart and aortic arc / carotid sinus stretch receptors, and of the central nervous system nuclei NTS and SPN:

$$\begin{array}{ccc} \Theta_{16}{\dot{x}}_{16}+x_{16} & = & f_{\tau_{1}}\left(x_{19}\right) \end{array}$$ (54)

$$\begin{array}{ccc} \Theta_{17}{\dot{x}}_{17}+x_{17} & = & g(x_{18}) \end{array}$$ (55)

$$\begin{array}{ccc} \Theta_{18}{\dot{x}}_{18}+x_{18} & = & f_{\tau_{3}}\left(x_{19}\right)+f_{\tau_{2}}\left(x_{20}\right) \end{array}$$ (56)

$$\begin{array}{ccc} \Theta_{19}{\dot{x}}_{19}+x_{19} & = & f(x_{1}) \end{array}$$ (57)

$$\begin{array}{ccc} \Theta_{20}{\dot{x}}_{20}+x_{20} & = & f(x_{1}) \end{array}$$ (58)

where *x*_1_ is the external input and *x*_16_ and *x*_17_ are the outputs.

Remarkably, the dynamical system formed by Eqs (39)–(58) can be seen as the negative feedback loop of an input-output monotone subsystem, formed by Eqs (39)–(53) (smooth muscle cell subsystem), where three distinct loops coexist, due to the effect of: (i) ANP released by the heart myocytes, stimulated by right atrium stretch receptors; (ii) AVP released by SPN, stimulated by right atrium stretch receptors; (iii) AVP released by SPN, stimulated by aortic arc / carotid sinus stretch receptors.

#### The AAV subsystem is monotone

**Fig 7:**
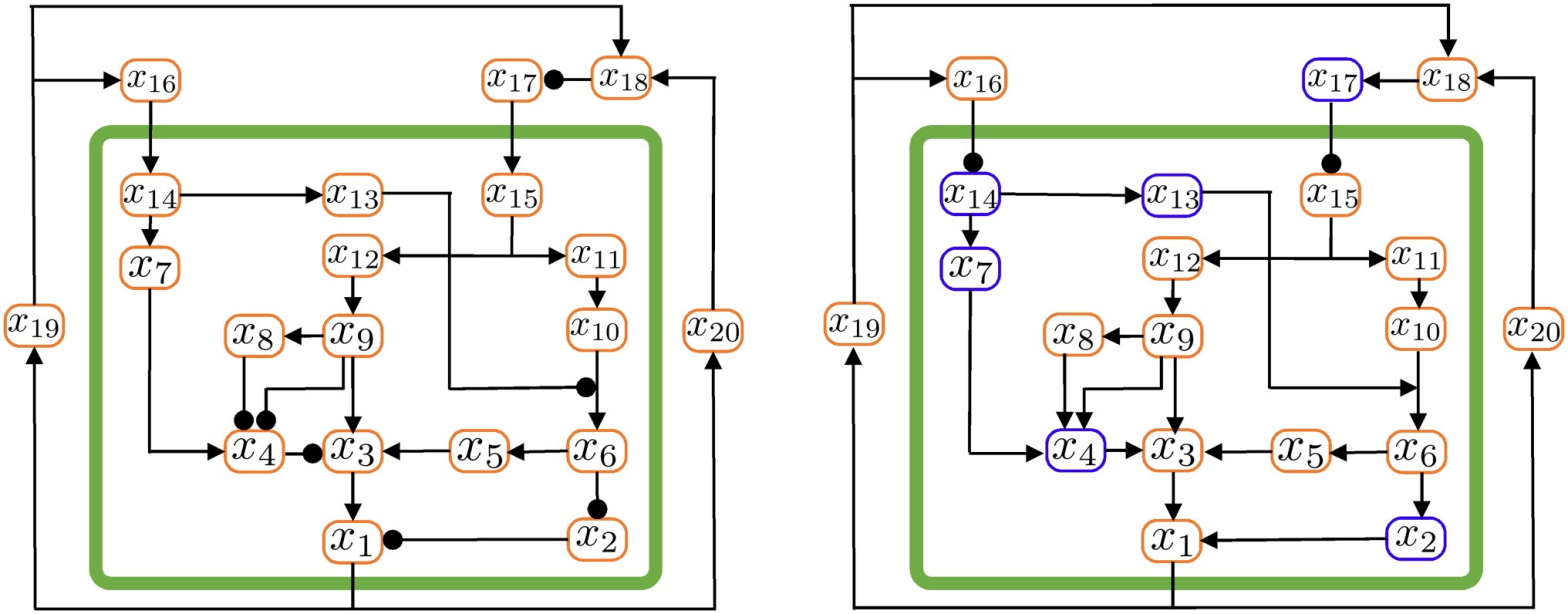
The interaction graph in Fig 3 (left) and its modified version (right). Pointed arrows represent activation arcs, while circle-head arrows represent inhibition arcs.

Consider the equivalent graph of the system in Fig 7, left, where the nodes represent variables and the arcs represent interactions. A path is a sequence of arcs connecting a starting node to a final node, passing through several intermediate nodes. A path is negative if it contains an odd number of negative (inhibitory) arcs. A loop is a closed path, where the starting node is the same as the final node.

We can change the sign of some variables in the system, associated with the graph nodes, so that inhibitory arcs become activating, and vice versa [61]: if the variable associated with a node changes sign, then all the arcs entering and leaving the node change their sign (activating or inhibitory) as well.

Changing sign to the six variables *x*_2_, *x*_4_, *x*_7_, *x*_13_, *x*_14_, *x*_17_ (associated with the nodes represented in blue in Fig 7, right) and sequentially changing the associated arc types yields the equivalent graph in Fig 7 (right). There, we see that the subsystem included in the box only contains activating arcs, meaning that all the variables are cooperating: it is a cooperative system.

Since the original subsystem included in the green box in Fig 7, left, is equivalent to a cooperative system by means of a state transformation where the sign of some variables is changed, it is a monotone system [60–65].

**Proposition 3** *The AAV subsystem formed by* Eqs (39)–(53)

1. *is a monotone system and has no internal loops*;
2. *has two inputs*, *x*_16_ *(associated with the activity of HMY releasing ANP) that inhibits the output x*_1_, *and x*_17_ *(associated with the activity of SPN releasing AVP) that activates x*_1_;
3. *has asymptotically stable equilibrium points: for any constant value of x*_16_ *and x*_17_, *all state variables converge to a steady-state value*.

In fact, any linearisation of this monotone system has a dominant negative real eigenvalue, which characterises its evolution and guarantees asymptotic stability.

#### All loops are negative

The inspection of the diagram in Fig 3, or equivalently in Fig 7, allows us to state the following fact.

**Proposition 4** *All the loops in the system formed by* Eqs (39)–(58) *are negative and include variable x*_1_ *(AMI)*.

Hence, the overall system is a candidate oscillator [15, 16]. This implies that it admits a single equilibrium point (when all the variables are at steady state); if the equilibrium becomes unstable due to the effect of the external loops, then oscillations occur.

In particular, the overall system can be seen as the feedback of the monotone subsystem, formed by the variables in the smooth muscle cell compartment, with three distinct negative feedback loops. Indeed, variable *x*_16_ has an inhibitory effect on *x*_1_, while *x*_17_ has an activating effect on *x*_1_; *x*_1_ has an activating effect on *x*_16_ and an inhibitory effect on *x*_17_.

Since all the elements in the smooth muscle cell subsystem evolve on a faster time scale with respect to those in the external loops, it is reasonable to approximate the whole subsystem given by Eqs (39)–(53) with a single variable *x*_1_, which evolves as a first order process with inputs *x*_16_ and *x*_17_:

$$\begin{array}{c} \Theta_{1}{\dot{x}}_{1}+x_{1}=g\left(x_{16}\right)+f\left(x_{17}\right) \end{array}$$ (59)

Moreover, since the effect of the delays *τ*_*i*_ strongly dominates with respect to the time constants also for the dynamics of the nodes in the external negative loops, the external connections can be seen as delayed static effects:

$$\begin{array}{ccc} x_{16} & = & f_{\tau_{1}}(x_{1}) \end{array}$$ (60)

$$\begin{array}{ccc} x_{17} & = & g_{\tau_{2}}(x_{1})+g_{\tau_{3}}(x_{1}) \end{array}$$ (61)

Hence, *x*_16_ and *x*_17_ are functions of delayed values of *x*_1_.
